# Computational identification and analysis of CNP0269688 as a natural product inhibitor disrupting the interaction between the HIV matrix domain and tRNA

**DOI:** 10.3389/fchem.2024.1450339

**Published:** 2024-09-02

**Authors:** Chengjie Xu, Songtao Wu, Pengju Liu, Yao Huang, Yuchao Chen, Guoping Ding, Shengnan Jia

**Affiliations:** ^1^ Department of General Surgery, Sir Run Run Shaw Hospital, School of Medicine, Zhejiang University, Hangzhou, China; ^2^ School of Medicine, Zhejiang University, Hangzhou, China; ^3^ Zhejiang Engineering Research Center of Cognitive Healthcare, Sir Run Run Shaw Hospital, School of Medicine, Zhejiang University, Hangzhou, China

**Keywords:** HIV-MA domain, protein-tRNA interactions, virtual screening, admet, MD simulation

## Abstract

Our research is dedicated to combating HIV by targeting its Matrix (MA) domain, which is crucial for viral assembly and replication. This strategy specifically aims to interrupt early-stage infection and deter drug resistance by focusing on this essential domain. Due to the MA domain’s conservation across different HIV strains, our approach promises broad-spectrum efficacy, which is particularly crucial in regions marked by significant genetic diversity and resistance issues. In our study, we introduce CNP0269688, a natural product that exhibits high affinity for the HIV-1 Matrix. Through detailed molecular dynamics simulations, we have assessed the compound’s structural stability and interaction dynamics, particularly its potential to hinder Protein-tRNA interactions. This analysis lays the groundwork for future experimental investigations. Our efforts are steps toward enhancing HIV treatment, reducing viral transmission, and curbing drug resistance, with the ultimate aim of controlling and eradicating the pandemic, thereby contributing significantly to public health and scientific advancement.

## 1 Introduction

Human Immunodeficiency Virus (HIV) continues to pose a significant global health challenge, affecting millions worldwide (Payagala and Pozniak, 2024). It is primarily transmitted through unprotected sexual intercourse, contaminated blood transfusions, and needle-sharing among intravenous drug users (Zhong et al., 2018). Advances in antiretroviral therapies have transformed HIV from a fatal illness into a manageable chronic condition (Yavuz et al., 2018). Nonetheless, current therapies, such as reverse transcriptase inhibitors, predominantly target the later stages of the HIV lifecycle (Li et al., 2022a). These treatments encounter several challenges, including drug resistance attributed to the high mutation rate of HIV, adverse effects, and elevated costs (Sever et al., 2024). This situation highlights the critical need for novel therapeutic strategies that target diverse aspects of the virus’s lifecycle to provide more effective and sustainable management of HIV.

The Gag protein of HIV, essential for the virus’s lifecycle, coordinates the assembly, encapsulation, budding, and maturation of virions (Bell and Lever, 2013). Its domains—Matrix (MA), Capsid (CA), Nucleocapsid (NC), and p6 as well as two small peptides SP1 and SP2 (spacer peptides one and 2) linking respectively the CA-NC and NC-p6 domains—each fulfill a specific role in these processes (Mouhand et al., 2020). The MA domain is particularly crucial as it mediates the initial attachment of the Gag polyprotein to the host cell membrane and significantly influences subsequent viral assembly (Banerjee and Voth, 2023). Furthermore, the MA domain interacts with host cell tRNAs, specifically Lys3 tRNA, which acts as a primer for reverse transcription, initiating the conversion of viral RNA into DNA within the host cell (Bou-Nader et al., 2021). This pivotal early-stage role renders the MA domain an attractive target for therapeutic intervention.

In light of the complexities and challenges associated with targeting other domains of the HIV-1 Gag protein, like CA, it's evident that considerable hurdles remain. The CA domain, highly conserved across different strains of HIV, is critical for the virus’s life cycle, making selective targeting challenging without affecting host cell functions (Rossi et al., 2021). Moreover, the high genetic variability of HIV complicates the development of effective inhibitors for the CA domain, as rapid mutation can lead to the emergence of drug-resistant strains (Chen et al., 2024).

The Nucleocapsid (NC) and p6 domains of the HIV-1 Gag protein also present unique challenges and opportunities for drug targeting. The NC domain, essential for RNA binding and packaging, is highly conserved and plays a crucial role in the formation of mature viral particles (Ding et al., 2016). Any intervention targeting the NC must avoid disrupting the essential processes of viral replication to prevent severe side effects (Lu et al., 2021b). The p6 domain, though smaller, is pivotal in the late stages of the virus’s life cycle, specifically in the budding process (Freed, 2015). The p6 domain interacts with the host cell’s ESCRT machinery, facilitating the final separation of new virions from the host cell (Chen and Wang, 2024). Disruption of this interaction offers a potential target for therapeutic intervention; however, targeting this process must be finely tuned to prevent unintended impacts on the host’s cellular processes, which also rely on the ESCRT machinery (Li et al., 2020).

Given these challenges, the design of inhibitors targeting the MA domain of the Gag protein has several advantages. The MA domain’s primary function in anchoring the Gag polyprotein to the plasma membrane makes it a less mutable and hence more attractive target compared to CA, NC, and p6 (Gaines et al., 2018). Additionally, the MA domain’s role in membrane fusion and virus assembly presents unique, distinct interfaces for drug targeting, potentially allowing for the development of inhibitors that are both effective and have minimal off-target effects on host cells (Parent and Gudleski, 2011).

Thus, focusing on the MA domain for the development of inhibitors, considering its critical and somewhat unique role in the viral lifecycle, offers a promising avenue for therapeutic development, potentially overcoming some of the limitations faced by current treatments targeting later stages of HIV replication.

To further the development of MA-targeting inhibitors, we plan to employ virtual screening (Carlsson and Luttens, 2024) to identify promising candidates, leveraging computational models to simulate interactions between potential inhibitors and the MA domain. Subsequent ADMET analysis (Kawsar et al., 2024) will assess the drug-like properties of these compounds, ensuring they possess essential pharmacological attributes such as bioavailability and minimal toxicity. Molecular dynamics simulations (Adediwura et al., 2024) will then evaluate the binding stability of these compounds, providing insights into their interactions with the MA domain. Additionally, we will utilize enhanced sampling methods, such as replica exchange dynamics (Lazim et al., 2020), to explore the effects of compound binding on the interaction between tRNALys3 and the MA domain. This analysis will elucidate the implications of inhibitor binding on the structural and functional integrity of the MA domain, which is crucial for the HIV lifecycle. These computational techniques will streamline the screening and optimization of MA-targeting inhibitors, supporting the development of novel antiretroviral drugs with improved efficacy and safety profiles.

## 2 Methods

### 2.1 Proteins preparation

HIV-1 Matrix domain to human tRNALys3 structure (PDB code: 7MRL) (Bou-Nader et al., 2021) was downloaded from PDB and used for virtual screening. The Matrix domain was extracted and prepared using the Protein Preparation Wizard tool of Schrödinger suite (Schrödinger Release 2023–2). The preparation protocol involved water and cofactor removal, element labeling correction, hydrogen atom addition, bond order assignment, hydrogen bond optimization, and energy minimization using OPLS4 force field (Lu et al., 2021a). Prepared proteins were used for grid generation with the Glide module (Schrödinger Release 2023–2) using the Receptor Grid Generation panel. Grids (15 Å × 15 Å × 10 Å) were generated at the centroid of the predicted druggable site using SiteMap.

### 2.2 Identification of druggable pockets

SiteMap was utilized to explore druggable pockets, employing OPLS4 force field to estimate interaction energies of probes on a three-dimensional grid covering the Matrix domain. The grid consisted of a minimum of 15 site points per site. SiteMap ranked the identified sites based on two druggability scores: SiteScore and Dscore. These scores assess the site’s characteristics such as size, solvent exposure, hydrophobicity, hydrophilicity, and hydrogen bond capability. The top-ranked regions were selected for embedding hotspot residues at the Matrix-tRNA interface (Bou-Nader et al., 2021). The SiteMap results guided the identification of binding sites for docking calculations.

### 2.3 Dataset and library preparation

The COCONUT (COlleCtion of Open Natural ProdUcTs) database (https://coconut.naturalproducts.net/) is a free natural product database containing 407,270 unique natural products. The products are sorted based on their annotation level, starting with the best annotated ones. The library was prepared using Schrödinger’s LigPrep tool in Maestro. LigPrep applied the OPLS4 force field, optimized the structures, and added hydrogen atoms. Within LigPrep, Epik was used to assign likely protonation states at pH 7 ± 2 and tautomers to each molecule.

### 2.4 Structure-based virtual screening

In the pursuit of potential lead compounds against HIV, we leveraged a structure-based virtual screening technique using the Glide algorithms. The process involved high-throughput virtual screening (HTVS), standard precision (SP), and extra precision (XP) levels. Initially, the best performing 10% from the HTVS outcomes were shortlisted and subsequently promoted to Glide SP. In a similar manner, the top tier 10% from the SP outcomes were retained and propelled to Glide XP. Ultimately, the highest-performing 10% from the XP outcomes were selected. In addition, Prime Molecular Mechanics–Generalized Born Model and Solvent Accessibility (MM-GBSA) values were computed for the indole derivative compounds, which enabled the ranking and pinpointing of the top ten compounds exhibiting the most favorable scores.

### 2.5 Binding pose metadynamics

The BPMD protocol was used for stability evaluation, using Monte Carlo (GCMC) to address water sampling (Ross et al., 2020). RMSD of ligand heavy atoms was the collective variable in ten 10 ns metadynamics simulations (Clark et al., 2016), each set is repeated 10 times. Hills had a height of 0.05 kcal/mol and width of 0.02 Å. RMSD calculation involved a 3 Å selection between protein and ligands. Prior to metadynamics, system preparation in an SPC water box took place, slowly reaching 300 K. The last 0.5 ns of the unbiased MD was used as a metadynamics reference. Three BPMD scores assessed ligand-binding stabilities. PoseScore reflects RMSD average (Fusani et al., 2020), PersScore measures hydrogen bond persistence, and CompScore combines both, with lower values indicating more stability (Jin et al., 2023).

### 2.6 Molecular dynamic simulation

MD simulations were executed using Schrödinger’s Desmond, starting with docked complexes in a 10 Å water buffer. Environments used SPC water and 0.15 M NaCl to replicate physiological conditions. Surplus ions maintained neutrality, while the particle-mesh Ewald technique handled long-range electrostatic interactions. Sequential simulation protocol followed, including Brownian Dynamics and NVT simulations at 10 K with restraints on solute heavy atoms. Different stages included various restrictions and temperatures, culminating in an NPT simulation at 300 K and 1 atm pressure.

### 2.7 ADMET prediction

Identified as leading candidates *via* structure-oriented virtual screening, the compounds were subsequently assessed through ADMETlab 3.0 (https://admetlab3.scbdd.com/). This evaluation sought to elucidate the pharmacokinetic profiles, bioavailability, and general appropriateness of these compounds for potential therapeutic use.

### 2.8 Replica exchange molecular dynamics

Replica exchange molecular dynamics(REMD) were executed using Schrödinger’s Desmond. The REMD temperature range is from 300 K to 500 K. The 40 replicas and 100 ns trajectory for each replica were collected. The exchange rate was fixed to 4 ps. To analyse the data, only the convergent trajectories at 300 K were employed.

## 3 Results

### 3.1 Docking studies of compounds with HIV-1 matrix

**FIGURE 1:**
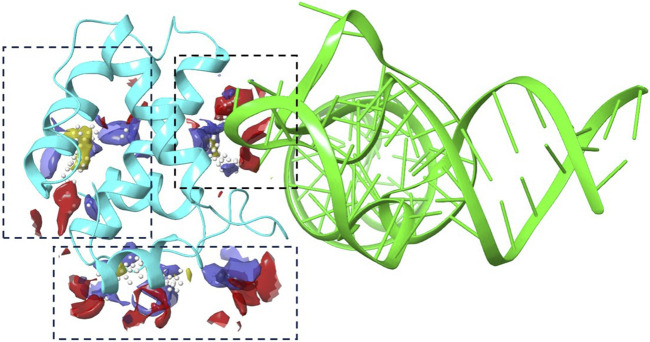
The binding site analysis results using Schrodinger’s SiteMap. A cartoon representation of tRNA (green) and protein (cyan) with various blue dashed boxes highlighting the pockets identified by Sitemap.

Employing the SiteMap for docking exploration, as illustrated in Figure 1, we identified three promising compounds likely to target the binding cavity. Notably, the second-ranked pocket, which includes amino acids Trp-16, Leu-21, Arg-22, Lys-27, Gln-28, Tyr-29, Lys-32, His-33, Ile-34, Trp-36, Ala-37, Glu-40, Glu-74, Arg-76, Ser-77, Leu-78, Asn-80, Ile-81, and Tyr-84, coincided with the tRNALys3 engagement region depicted in the crystal structure. This finding prompted its designation as the primary focus area for subsequent virtual screening efforts.

Following the completion of a multi-phase virtual screening process, we amassed a collection of 240 candidate compounds. To minimize the occurrence of false positives, we computed the binding free energies (∆G_bind) for each ligand-protein complex. Among these candidates, we selected 12 compounds that exhibited ∆G_bind values below −40 kcal/mol and demonstrated minimal ligand strain energy (less than 5.0 kcal/mol). These compounds underwent a comprehensive evaluation of their docking poses within the HIV-1 Matrix binding cavity. Detailed XP GScore, MMGBSA ∆G_bind, and Ligand Strain Energy metrics for these compounds are presented in Table 1.

**TABLE 1:** Evaluation results of drug compounds.

Compound ID	XP GScore	Glide ligand efficiency	MMGBSA dG bind (Kcal/mol)	Lig strain energy (Kcal/mol)
CNP0128162	−6.854	−0.263	−52.4	4.92
CNP0293279	−7.58	−0.271	−47.55	1.672
CNP0410458	−7.01	−0.3	−45.29	4.892
CNP0331343	−7.747	−0.298	−43.2	3.586
CNP0269688	−8.137	−0.291	−42.69	1.708
CNP0091610	−7.006	−0.275	−41.99	2.013
CNP0001676	−8.903	−0.342	−41.36	4.472
CNP0227699	−6.745	−0.281	−41.16	2.534
CNP0074439	−8.136	−0.301	−40.72	4.985
CNP0375461	−6.66	−0.247	−40.71	2.573
CNP0473555	−6.618	−0.276	−40.59	2.546
CNP0113308	−7.376	−0.238	−40.28	4.104

**FIGURE 2:**
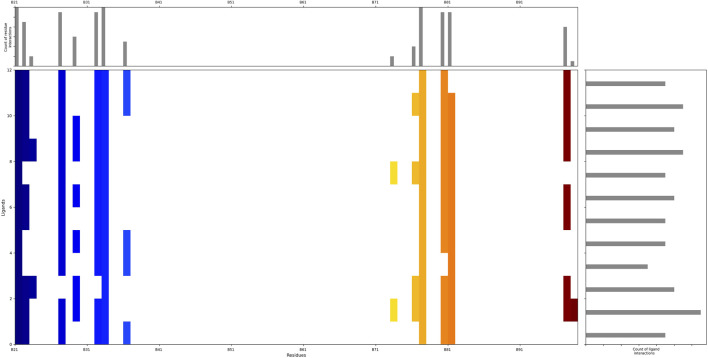
The interaction fingerprint matrix of docking poses into the HIV-1 Matrix site. Colored bars indicate one or more interactions between HIV-1 Matrix residues (columns) and individual ligands (rows). Diferent colors indicate diferent residues.

**FIGURE 3:**
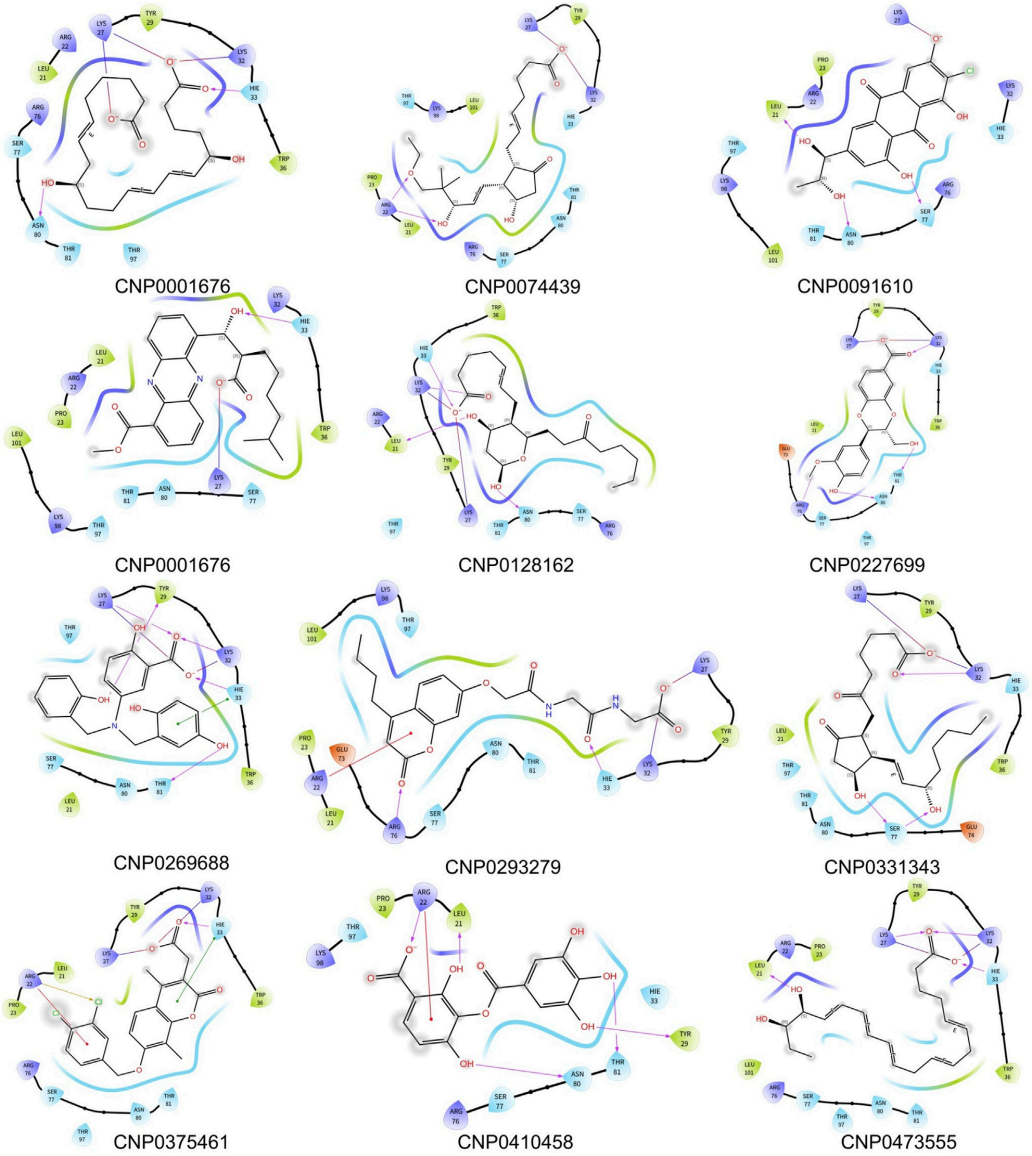
Binding pattern diagrams of retained compounds.

To investigate the specific residues within the engagement site that interact with the shortlisted 12 compounds, we utilized Maestro to generate an interaction fingerprint (IFP) matrix, as shown in Figure 2. Setting a cutoff value of one for the count of residue interactions, it was determined that Leu-21, His-33, and Ser-77 of the HIV-1 Matrix participated in interactions with all 12 compounds. Additional residues, including Arg-22, Lys-27, Lys-32, Tyr-79, Asn-80, Thr-81, and Thr-97, were associated with more than half of the compounds. In contrast, Pro-23, Tyr-29, Trp-36, Glu-73, and Lys-98 exhibited notable selectivity, engaging with only a limited subset of compounds, as detailed in Figure 3.

### 3.2 Conformation evaluation of composite based on BPMD

**FIGURE 4:**
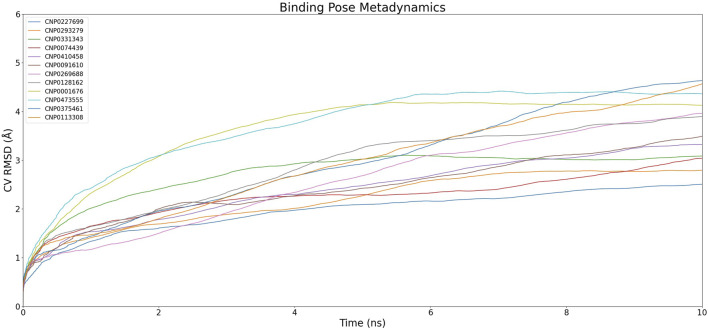
Dynamics of Complexes' CV RMSD over Simulation Time.The graph illustrates the variation of CV RMSD (Collective Variable Root Mean Square Deviation) over time for different compounds. Each color represents a different compound and its corresponding complex, showing the dynamics and fluctuations of the complexes throughout the simulation.

To ensure rigorous and precise determination of the ligand docking positions, the docking results for the compounds were assessed using Brownian Post-Molecular Dynamics (BPMD) simulations to evaluate their reliability. The mean root-mean-square deviation (RMSD) values for the ligands' heavy atoms, calculated across ten iterations, were plotted against simulation time as depicted in Figure 4. The peak RMSD value on each curve corresponds to the PoseScore value for the respective pose. Additionally, the PoseScore, Persistence Score (PersScore), and Composite Score (CompScore) for each ligand are comprehensively detailed in Table 2. This dataset facilitates the classification and analysis of the compounds’ characteristics, allowing them to be grouped into distinct clusters based on their PoseScore, PersScore, and CompScore values.

**TABLE 2:** Comparative scores of compound ID for binding poses.

Compound ID	CNP0227699	CNP0293279	CNP0331343	CNP0074439	CNP0410458	CNP0091610	CNP0269688	CNP0128162	CNP0001676	CNP0473555	CNP0375461	CNP0113308
PoseScore	2.438	2.779	3.032	2.818	3.211	3.28	3.776	3.772	4.141	4.379	4.455	4.222
PersScore	0.178	0.151	0.185	0.048	0.109	0.1	0.149	0.105	0.085	0.127	0.095	0.009
CompScore	1.548	2.024	2.107	2.578	2.666	2.78	3.031	3.247	3.716	3.744	3.98	4.177

In the initial cluster, compounds CNP0227699, CNP0293279, and CNP0074439 are characterized by relatively low PoseScore and PersScore values, yet they exhibit moderate CompScore values, indicating acceptable structural consistency and overall stability. The second cluster includes CNP0331343, CNP0410458, and CNP0091610, which demonstrate higher PoseScore and PersScore values, yet maintain relatively high CompScore values, suggesting significant structural consistency and enhanced stability. The third cluster, comprising CNP0269688 and CNP0128162, displays increased PoseScore and PersScore values, along with high CompScore values, reflecting substantial structural consistency and robust stability. Lastly, the fourth cluster, consisting of CNP0001676, CNP0473555, and CNP0375461, features the highest PoseScore values, albeit with somewhat lower PersScore values. Nevertheless, these compounds still achieve relatively high CompScore values, indicative of commendable structural consistency and overall stability.

### 3.3 Molecular dynamic simulation primary screening of structural stability

**FIGURE 5:**
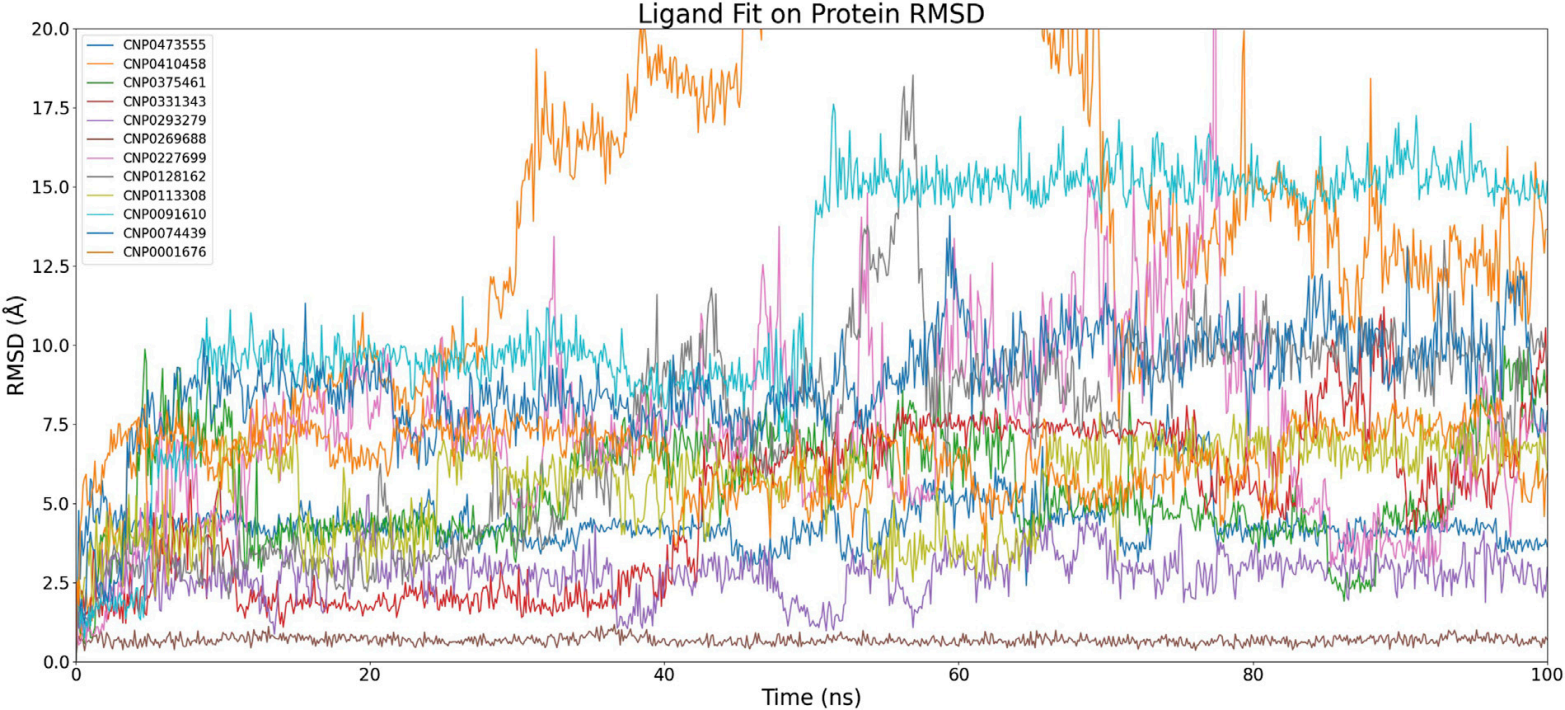
Time-dependent RMSD of Compound Fit on Protein Complexes. The graph depicts the changes in RMSD (Root Mean Square Deviation) of ligand fit on protein complexes over simulation time. Each color corresponds to a different compound and showcases the fluctuations in ligand-protein complex conformations throughout the simulation.

To ascertain the precise binding modality and evaluate the stability of the complexes, comprehensive molecular dynamics (MD) simulations were utilized to assess the durability of protein-ligand interactions. The stability of these twelve candidate compounds was rigorously analyzed by calculating the ligand fit onto protein root-mean-square deviations (RMSDs), as depicted in Figure 5. These calculations provided insights into the complex dynamics governing the protein-ligand interactions.

After discarding compounds that demonstrated considerable instability during the simulations or exhibited significant RMSD variations, a select pool of four compounds was chosen for further scrutiny: CNP0473555, CNP0293279, CNP0269688, and CNP0091610. Within this subset, both CNP0091610 and CNP0269688 reached a stabilization phase almost concurrently. While CNP0091610 displayed persistent RMSD oscillation of 2.5 Å post-stabilization, this may be attributed to the inherent flexibility of the molecule, indicative of the initial formation of a stable bond with the HIV-1 Matrix. Conversely, CNP0293279 did not exhibit a discernible stabilization phase; however, its maximum RMSD remained below 4 Å, indicating only minor conformational shifts compared to the initial structure throughout the simulation. Notably, RMSD fluctuations for CNP0293279 were consistently smaller than those observed for CNP0091610, despite CNP0091610 eventually achieving a stabilization phase.

Additionally, the analysis of CNP0473555 revealed intermittent rises and falls in RMSD within the 50–80 ns range. Nevertheless, given that the RMSD predominantly remained within the 4–5 Å range for the majority of the simulation, CNP0473555 was retained among the four compounds for further in-depth exploration. This meticulous selection process underscores the importance of dynamic stability and minimal conformational variability in the potential therapeutic efficacy of these compounds against HIV.

### 3.4 Molecular dynamic simulation rescreening of structural stability

To enhance the analysis of the selected compounds, a rigorous molecular dynamics simulation protocol was implemented, involving 300 ns simulations, each executed in triplicate to enhance statistical robustness. This extended simulation duration was designed not only to deepen our understanding of their molecular behaviors but also to verify the reproducibility of the results. The primary objective was to elucidate the complex dynamics of these compounds and assess their potential for future therapeutic applications.

**FIGURE 6:**
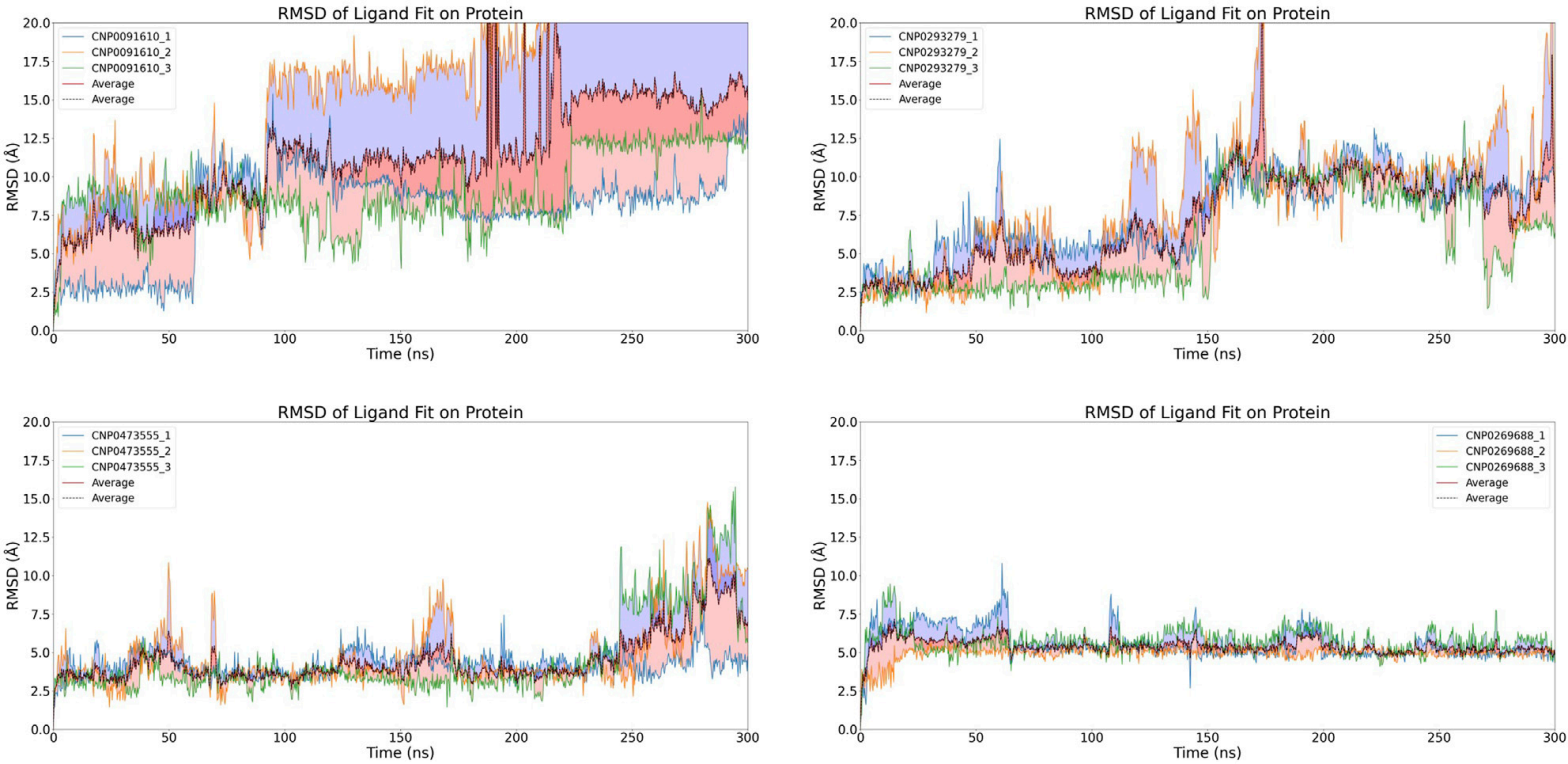
Time-dependent RMSD of Compound Fit on Protein Complexes with with Replicate Analysis.The graph displays the changes in RMSD of compound fit on HIV-1 Matrix protein complexes over simulation time. Each individual image represents a different compound forming a complex with HIV-1 Matrix, and different-colored curves within each image depict replicate results. The dashed line indicates the average RMSD value from three independent replicates.

The results, illustrated in Figure 6, reveal significant variability among the three independent simulations of CNP0091610, indicating inconsistent binding to the HIV-1 Matrix. Similarly, the three simulations of CNP0293279 exhibited dynamics comparable to those of the previously discarded eight compounds, with the RMSD failing to stabilize, suggesting an unstable attachment to the HIV-1 Matrix.

For CNP0473555, one subset exhibited significant RMSD fluctuations during the initial 250 ns, while the other simulations achieved a stable equilibrium. However, in the concluding 50 ns, all subsets displayed varying levels of RMSD escalation, pointing to a temporal dependency in binding stability and raising concerns about the potential for non-specific interactions.

Conversely, CNP0269688, after significant initial conformational adjustments, demonstrated more consistent behavior; only a few replicates showed minor fluctuations, while the majority exhibited notable stability. Considering the inherent complexity and diversity of natural product conformations, these observations are deemed acceptable. Thus, after extended simulation, CNP0269688 emerges as the only compound demonstrating consistent and stable affinity to the HIV-1 Matrix, highlighting its potential as a viable candidate for further therapeutic development.

### 3.5 Physicochemical and ADMET properties of CNP0269688

In this study, we conducted a comprehensive computational analysis of compound CNP0269688, focusing on its ADMET (Absorption, Distribution, Metabolism, Excretion, and Toxicity) properties to evaluate its viability as a drug candidate. This detailed assessment offers valuable insights into the compound’s pharmaceutical potential.

Compound CNP0269688 has a molecular weight (MW) of 380.37 g/mol and comprises 28 heavy atoms, 18 of which are aromatic. With a fraction Csp3 of 0.1 and six rotatable bonds, this compound demonstrates moderate structural complexity. It features six hydrogen bond acceptors and four hydrogen bond donors, indicative of its potential for robust interactions with biological targets.

Solubility is a critical parameter in drug development. Predictive models suggest moderate solubility for CNP0269688: ESOL Log S (−4.6), Ali Log S (−5.99), and Silicos-IT LogSw (−4.59) imply limited aqueous solubility. Quantitatively, ESOL predicts a solubility of 9.49E-03 mg/mL (2.50E-05 mol/L), Ali at 3.89E-04 mg/mL (1.02E-06 mol/L), and Silicos-IT at 9.80E-03 mg/mL (2.58E-05 mol/L). These findings suggest that formulation strategies may be necessary to enhance the compound’s solubility for oral administration.

Analyzing the compound’s ADME properties is essential to evaluate its potential for drug development. CNP0269688 exhibits high gastrointestinal (GI) absorption, which is beneficial for oral administration. However, it is not expected to cross the blood-brain barrier (BBB) or act as a P-glycoprotein (Pgp) substrate, suggesting limited central nervous system (CNS) activity and reduced susceptibility to Pgp-mediated efflux.

CNP0269688 complies with several pharmaceutical guidelines, including Lipinski’s Rule of Five, Ghose’s Rule of Five, Veber’s Rule, Egan’s Rule, and Muegge’s Rule, with no violations noted. However, the presence of alerts related to pan-assay interference compounds (PAINS) and leadlikeness violations necessitates further evaluation of its suitability as a drug candidate. The synthetic accessibility score of 2.35 indicates that synthesis is feasible within typical laboratory settings.

In summary, CNP0269688 presents several promising attributes, such as high GI absorption and conformity to key physicochemical rules. Nonetheless, its limited aqueous solubility and potential inhibitory effects on certain cytochrome P450 enzymes require careful consideration. While computational analyses provide valuable preliminary insights, experimental validation is imperative to confirm these predictions and further assess the compound’s safety, efficacy, and potential for drug-drug interactions. Subsequent research and development efforts are essential to fully determine its viability as a pharmaceutical agent.

### 3.6 Protein-ligand interactions analysis

**FIGURE 7:**
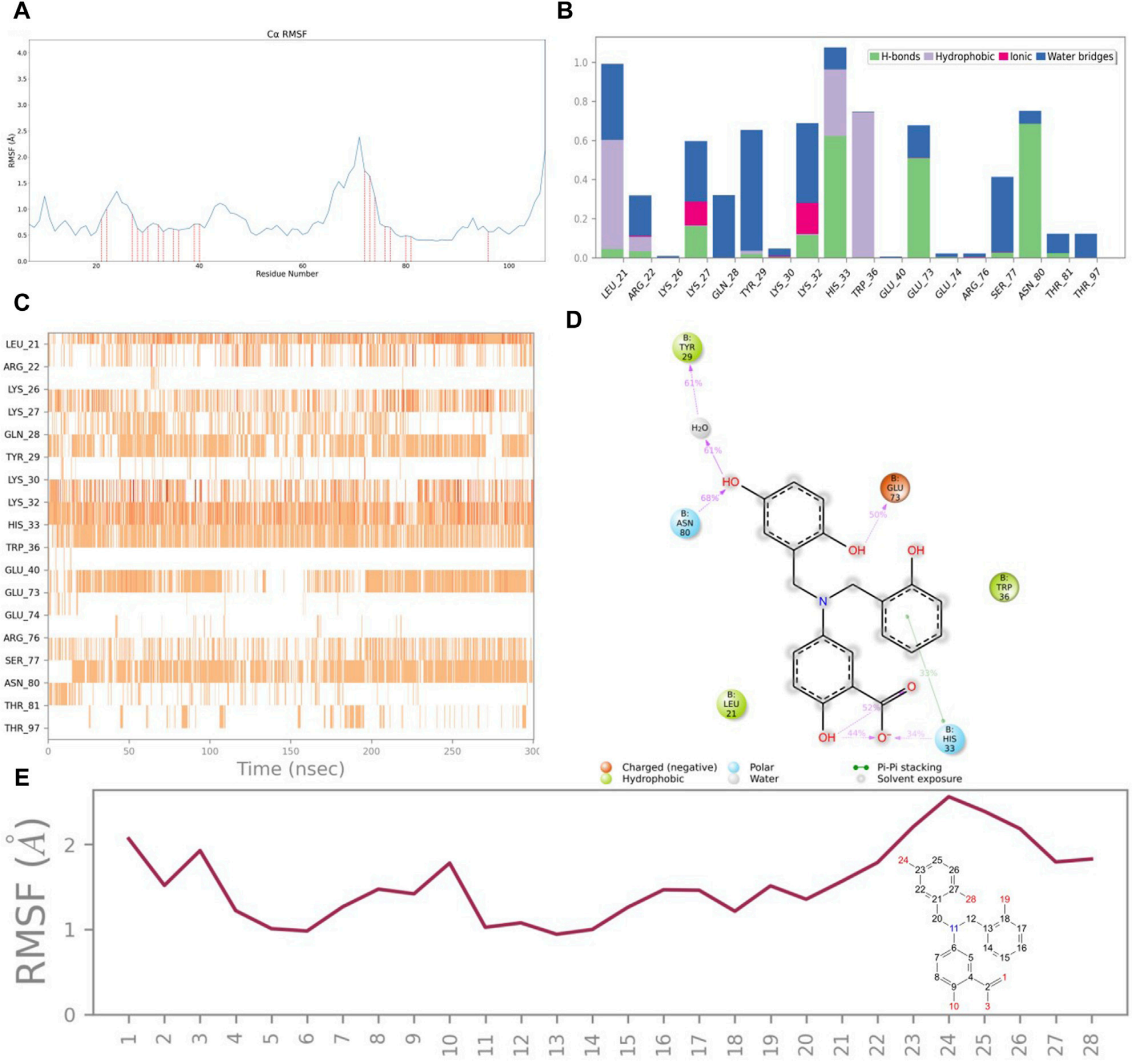
Protein and ligand interactions analysis. **(A)** RMSF of HIV-1 Matrix. **(B)** HIV-1 Matrix interactions with CNP0269688 throughout the simulation. **(C)** Timeline representation of HIV-1 Matrix-CNP0269688 interactions. **(D)** Schematic diagram showing the detailed atomic interactions of HIV-1 Matrix with CNP0269688. **(E)** RMSF of CNP0269688.

To evaluate the structural association between CNP0269688 and the HIV-1 Matrix, a Root Mean Square Fluctuation (RMSF) analysis was performed, the results of which are depicted in Figure 7A. The analysis identified 18 residues in direct contact with the compound, including Leu-21, Arg-22, Lys-26, Lys-27, Gln-28, Tyr-29, Lys-30, Lys-32, His-33, Trp-36, Glu-40, Glu-73, Glu-74, Arg-76, Ser-77, Asn-80, Thr-81, and Thr-97. Notably, a subset of these residues—Arg-22, Lys-27, Lys-32, His-33, Trp-36, Arg-76, and Ser-77—are known to interact with tRNA, suggesting potential competition between CNP0269688 and tRNA for binding sites on the HIV-1 Matrix.

Further investigations into the interaction dynamics between CNP0269688 and these residues were explored through the creation of PL-Contacts plots (Figures 7B, C). Arg-76 engages in transient, minimal hydrophobic and ionic interactions with CNP0269688. Meanwhile, residues such as Arg-22, Lys-27, Lys-32, His-33, Trp-36, Arg-76, and Ser-77 engage in three distinct types of interactions with the compound. His-33 is notably involved in water bridging, hydrophobic interactions, and hydrogen bonding. The remaining residues interact with CNP0269688 through mechanisms including water bridging, ionic interactions, and hydrogen bonding, with Arg-22 exhibiting a diverse array of connections such as water bridging, hydrophobic interactions, ionic interactions, and hydrogen bonding. Despite a higher interaction frequency with Arg-22 compared to Arg-76, noticeable differences are observed in the interaction frequencies with Lys-27, Lys-32, His-33, Trp-36, and Ser-77. Hydrogen bonding and water bridging play a crucial role in maintaining the stable attachment of CNP0269688 to the HIV-1 Matrix.

To elucidate the structure-activity relationship, an LP-Contacts Summary plot was generated (Figure 7D), highlighting interactions between CNP0269688 and the protein that persisted for at least 30% of the simulation time. This plot reveals specific functional group interactions within the HIV-1 Matrix, including the carboxyl group at position 4, the phenolic group at position 12, the hydroxyl group at position 23, and the phenolic hydroxyl group at position 27.

The Ligand RMSF analysis (Figure 7E) indicated that the thermal movement of benzene rings near His-33 is constrained by the formation of hydrogen bonds with the carboxyl group at position four and π-π stacking interactions with the phenolic group at position 12. Conversely, the catechol moiety, which forms water bridges and hydrogen bonds with Tyr-29, Glu-73, and Asn-80, exhibits greater flexibility.

**FIGURE 8:**
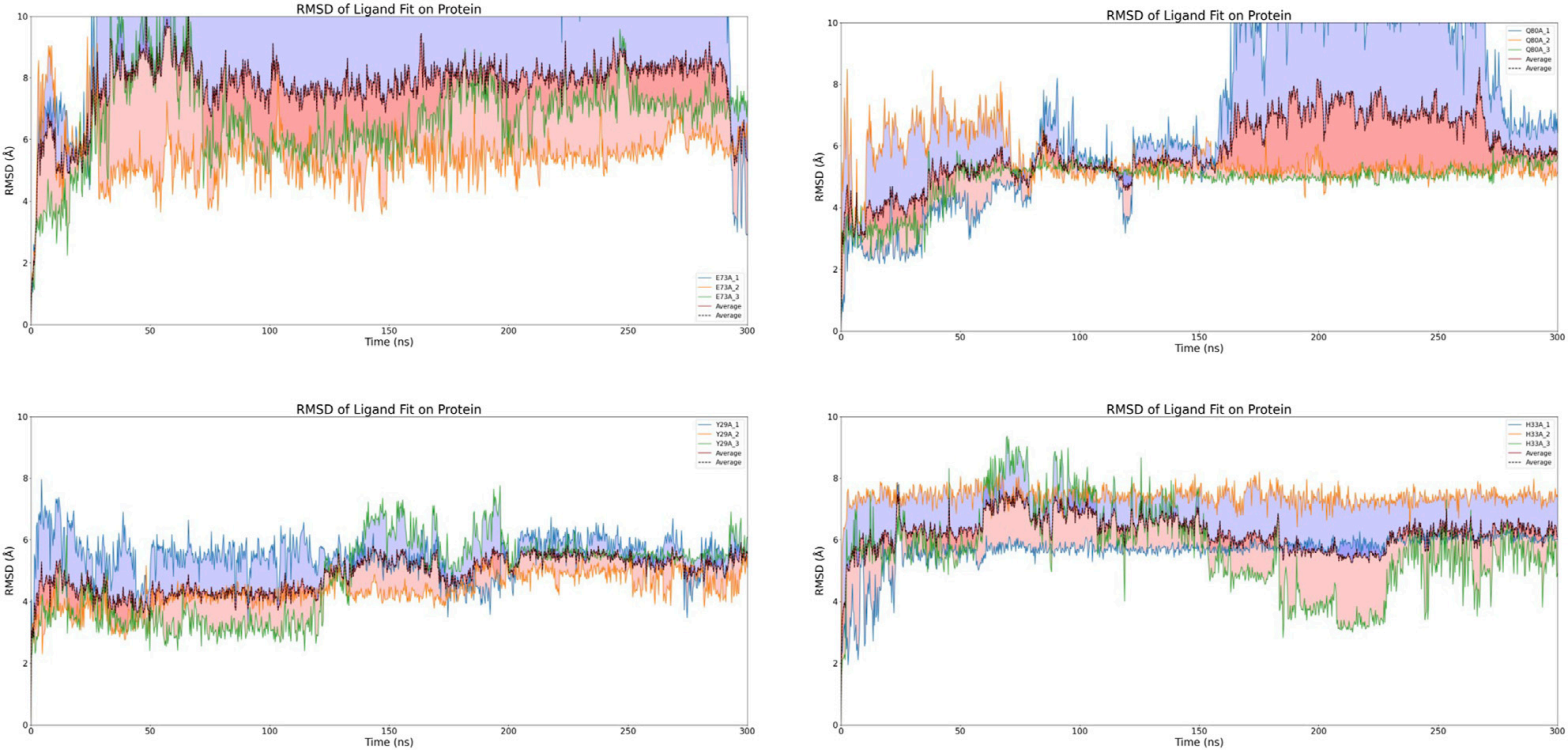
RMSD of HIV-1 Matrix mutants.

To confirm the structure-activity relationship between CNP0269688 and the HIV-1 Matrix, alanine mutagenesis was performed on the four key residues. The RMSD findings from this experiment showed that compounds binding to the mutated residues were less stable than those binding to the wild-type (WT) HIV-1 Matrix-CNP0269688 complex, as shown in Figure 8, thereby validating the trajectory analysis results. This rigorous approach strongly supports the reliability of our findings and provides a robust foundation for further pharmaceutical exploration.

### 3.7 Functional validation of CNP0269688 as a protein-tRNA interaction inhibitor through enhanced sampling

**FIGURE 9:**
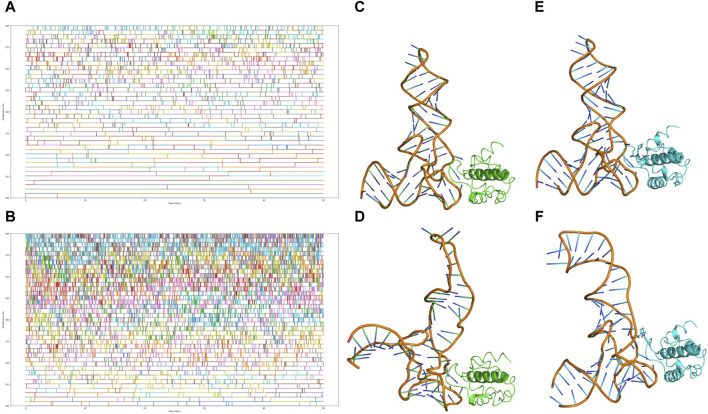
Analysis of replica exchange frequency and conformational changes. **(A)** Copy exchange records of HIV-MA-tRNA complexes during the simulation process. **(B)** Copy exchange records of HIV-MA-tRNA CNP0269688 complex during the simulation process. **(C)** The initial conformation of the HIV-MA-tRNA complex. **(D)** The conformation of the HIV-MA-tRNA complex after 100 ns REMD. **(E)** The initial conformation of the HIV-MA-tRNA CNP0269688 complex. **(F)** The conformation of the HIV-MA-tRNA CNP0269688 complex after 100 ns REMD.

In our subsequent investigations, we conducted two sets of Replica Exchange Molecular Dynamics (REMD) simulations to further explore the impact of CNP0269688 on the interactions between HIV-MA and tRNA. These simulations were executed with 40 replicas, with temperatures ranging evenly from 300 K to 500 K, and each simulation extended over 100 ns. The outcomes of these simulations are presented in Figures 9A, B. In both sets of REMD simulations, frequent replica exchanges were consistently observed, particularly exhibiting a high exchange rate when CNP0269688 was integrated into the system. This phenomenon is indicative of an accelerated exploration of the energy landscape, facilitating more rapid equilibration within the simulation. Additionally, the increased frequency of replica exchanges enhances the diversity of sampling, which diminishes the likelihood of the simulation becoming confined to specific conformations or states. Consequently, it is plausible to assert that the REMD simulations with CNP0269688 binding achieved comprehensive and diverse sampling.

Further trajectory analysis involved a comparative evaluation of conformational changes in the complex before and after the simulation, as depicted in Figures 9C–F. As the simulation progressed, a trend was observed where tRNA incrementally approached HIV-MA in scenarios where it was not bound to HIV-MA, a configuration typically conducive to the functioning of HIV-MA. In stark contrast, when HIV-MA was bound to CNP0269688, tRNA displayed divergent behavior, progressively distancing itself from HIV-MA. Based on these dynamic observations, there are compelling reasons to propose that CNP0269688, when targeting HIV-MA, may act as an inhibitor of the protein-tRNA interaction, potentially disrupting essential biological processes within the virus lifecycle. This characteristic underscores the potential therapeutic value of CNP0269688 as a modulator of protein-tRNA interactions in HIV.

## 4 Disucssion

The MA (Matrix) domain of the HIV Gag protein plays a critical role in the early stages of viral replication by facilitating v and budding (Jin et al., 2023). Inhibiting this domain has emerged as a promising strategy for developing promising therapeutics against HIV. By disrupting the interaction between the viral core and the host cell membrane during viral assembly, inhibitors targeting the MA domain can prevent the formation of infectious viral particles (Jin et al., 2023). Combining MA domain inhibitors with existing antiretroviral drugs in combination therapy can enhance treatment efficacy and overcome drug resistance, improving patient outcomes (Weichseldorfer et al., 2021). Studying the MA domain and its interactions with small molecule inhibitors provides valuable insights into the structural and functional aspects of viral assembly, enabling the design and optimization of next-generation inhibitors with improved properties (Zentner et al., 2013). By targeting the MA domain, it is possible to disrupt viral replication at an early stage, potentially reducing the emergence of drug-resistant strains. These advancements in inhibiting the HIV Gag MA domain offer great potential in the field of HIV therapeutics, providing opportunities to enhance current treatment strategies and develop more effective and long-lasting therapies against HIV/AIDS.

CNP0269688, also known as Lavendustin A, is a natural compound with potential anticancer and anti-inflammatory properties (Hu and Fan, 1995; Whitehead and Lacey, 2000; Zentner et al., 2013). In our study, we discovered that Lavendustin A exhibits high affinity and stable binding to the MA protein, specifically targeting Lys-32 and Trp-36. This interaction likely disrupts tRNA binding and redistributes Gag to the plasma membrane. Consequently, the impaired ability of Gag to bind tRNA results in reduced HIV-1 replication.

Through analysis of the dynamic trajectory of CNP0269688, it was observed that the hydroxyl groups on the catechol moiety can form hydrogen bonds or water bridges with residues. However, the hydroxyl group on the phenol moiety does not participate in hydrogen bonding or water bridging but instead engages in π-π stacking interactions. In general, hydrogen bonds are stronger than π-π stacking interactions (Grover et al., 2021). Hydrogen bonds are strong intermolecular forces involving the interaction between a hydrogen atom and a more electronegative atom such as nitrogen, oxygen, or fluorine (Cai et al., 2022). They possess high binding energy and exhibit a relatively longer interaction distance, playing a crucial role in structural stability and functionality of biomolecules. On the other hand, π-π stacking interactions are weaker and involve interactions between π electron clouds (Molcanov and Kojic-Prodic, 2019). They commonly occur in aromatic rings or conjugated systems, contributing to stability within molecules or between molecules. However, compared to hydrogen bonds, π-π stacking interactions typically have shorter interaction distances and lower binding energies, resulting in less significant structural and functional effects (Deng et al., 2020; Li et al., 2022b). It should be noted that the strength of interactions is influenced by various factors including molecular geometry, charge distribution, solvent environment, among other (Khan and Brettmann, 2019). In certain specific cases, π-π stacking interactions may demonstrate comparable or even stronger intermolecular forces than hydrogen bonds (Deng et al., 2020). Based on the findings of this study, the thermal fluctuations of the phenolic group, which forms π-π stacking interactions with residues, are relatively restrained, whereas the catechol moiety, which forms hydrogen bonds or water bridges with residues, exhibits more pronounced thermal motions, potentially leading to off-target effects. Therefore, in further research, modifying the phenol moiety to a catechol moiety could enhance the binding stability by establishing new π-π stacking interactions with Tyr-29, thereby increasing the affinity between the compound and the target.

## 5 Conclusion

This study demonstrates CNP0269688 as a potent inhibitor of the HIV-1 Matrix protein, crucial in early HIV replication stages. Through molecular docking and dynamics simulations, we identified its potential to disrupt Matrix-tRNA interactions, a novel therapeutic approach targeting early viral assembly. This suggests CNP0269688 and similar natural compounds as promising candidates for reducing drug-resistant HIV strains. Future research will validate these findings experimentally and optimize derivatives for clinical use.

## Data Availability

The original contributions presented in the study are included in the article/Supplementary Material; further inquiries can be directed to the corresponding author.
